# Accuracy of Bone Measurements in the Vicinity of Titanium Implants in CBCT Data Sets: A Comparison of Radiological and Histological Findings in Minipigs

**DOI:** 10.1155/2017/3848207

**Published:** 2017-07-17

**Authors:** Alexander Gröbe, Jan Semmusch, Maximilian Schöllchen, Henning Hanken, Michael Hahn, Wolfgang Eichhorn, Gerhard Schön, Ole Jung, Jamal M. Stein, Aline Reitmeier, Max Heiland, Ralf Smeets, Clarissa Precht

**Affiliations:** ^1^Department of Oral and Maxillofacial Surgery, University Medical Center Hamburg-Eppendorf, Hamburg, Germany; ^2^Department of Osteology and Biomechanics, University Medical Center Hamburg-Eppendorf, Lottestraße 59, 22529 Hamburg, Germany; ^3^Department of Medical Biometry and Epidemiology, University Medical Center Hamburg-Eppendorf, Hamburg, Germany; ^4^Department of Operative Dentistry, Periodontology and Preventive Dentistry, RWTH Aachen University Hospital, Aachen, Germany; ^5^Department of Laboratory Animal Science, University Medical Center Hamburg-Eppendorf, Hamburg, Germany; ^6^Charité-Universitätsmedizin Berlin, Corporate Member of Freie Universität Berlin, Humboldt-Universität zu Berlin, and Berlin Institute of Health, Department of Oral and Maxillofacial Surgery, Berlin, Germany

## Abstract

**Purpose:**

The aim of this animal study was the determination of accuracy of bone measurements in CBCT (cone-beam computed tomography) in close proximity to titanium implants.

**Material and Methods:**

Titanium implants were inserted in eight Göttingen minipigs. 60 implants were evaluated histologically in ground section specimen and radiologically in CBCT in regard to thickness of the buccal bone. With random intercept models, the difference of histologic measurements and CBCT measurements of bone thickness was calculated.

**Results:**

The mean histological thickness of the buccal bone was 5.09 mm (CI 4.11–6.08 mm). The four raters measured slightly less bone in CBCT than it was found in histology. The random effect was not significant (*p* value 1.000). Therefore, the Intraclass Correlation Coefficient (ICC) was 98.65% (CI 100.00–96.99%).

**Conclusion:**

CBCT is an accurate technique to measure even thin bone structures in the vicinity of titanium implants.

## 1. Introduction

Cone-beam computed tomography (CBCT) was introduced in medical imaging in 1998 by Mozzo et al. [1]. While the authors intended this technique as a computed tomography device in dental imaging, CBCT has extended its indications ever since. In recent guidelines from 2013, the indications are formulated such as assessment of pathologies in dental root structures, preoperative trauma diagnostics, imaging of intraosseous lesions, and implantologic planning [2]. Apart from the head and neck region, there are possibilities of imaging of the female breast or picturing osseous lesions of the wrist [3, 4]. Compared to the multislice computed tomography (MSCT), there is a lack of soft tissue discrimination, but its resolution of high contrast structures provides diagnostic value, which can even exceed MSCT [5]. Even in detection of osseous invasion of oral squamous cell carcinoma, CBCT could be valuable [6]. Good image quality and a relatively low radiation exposure compared to MSCT are the benefits of this technique [7–12].

For a correct interpretation of the three-dimensional dimensions of anatomical and pathological structures in the head and neck region, an accurate acquisition and visualization are required to perform sufficient measurements. In this context, Guijarro-Martínez and Swennen showed that CBCT is suitable for measurements and analysis of the upper airway [13]. Friedrich et al. found CBCT suitable for the determination of orbital volumes, while other authors evaluated its imaging value in measurement of Schneiderian membrane thickness, visualization of apical periodontitis, and reliability of volume data sets using virtual models [14–17].

Even though artifacts can lower image quality to a certain extent, CBCT is generally considered to provide sufficient data for measurements of bone volume even in the vicinity of titanium dental implants [18–21]. The purpose of the present study was to evaluate this accuracy by using an animal model and comparing bone measurements of a thin buccal lamella of CBCT datasets with corresponding histological sections.

## 2. Materials and Methods

### 2.1. Animals

The laboratory animals were eight healthy, full-grown minipigs (Göttingen Minipig®) [22], aged 24 to 30 months. In addition to the already described similarities in bone physiology, they show good postoperative healing assets and a high indolence after surgery [23]. All animals used in this study were specifically bred for laboratory animal use (Ellegaard Göttingen Minipigs A/S, Denmark). The experimental project with the number 112/15 was approved by the animal welfare Commission of the Office for Health and Consumer Protection, Hamburg (Germany). The experiments were carried out according to the European Communities Council Directive of 24 November 1986 (86/609/EEC) and in accordance with German laws and regulations.

### 2.2. Surgical Procedure

A perioperative antibiotic prophylaxis was administered for 7 days (Unacid® 375 mg). Each animal was injected i.m. with a weight-dependent dose of ketamine 10%, xylazine 2%, and 3 ml of Stresnil™. Depending on the depth of sedation, 2–4 mg propofol 2% i.v. was administered. With the inhalation anesthetic, Isoflurane-Baxter 1.5%, the anesthetic was maintained. As volume replacement, Jonosteril® 1.5 l and HAES-sterile 0.5 l were infused during the procedure. To ensure a sufficient analgesia, a vestibular and oral local infiltration of 5 ml of Ultracain® 4% with adrenaline 1 : 100.000 was given.

**Figure 1:**
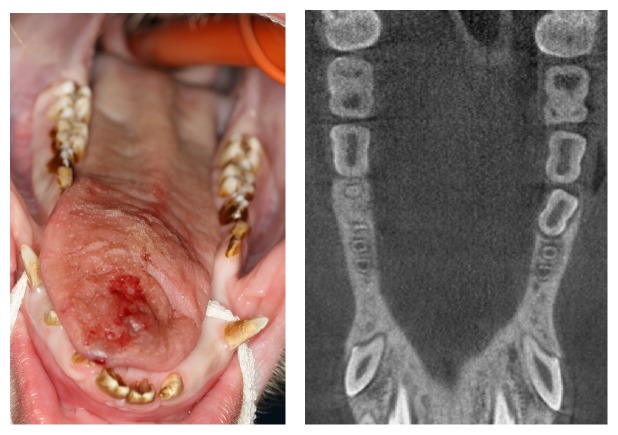
Clinical assessment and CBCT before surgery.

**Figure 2:**
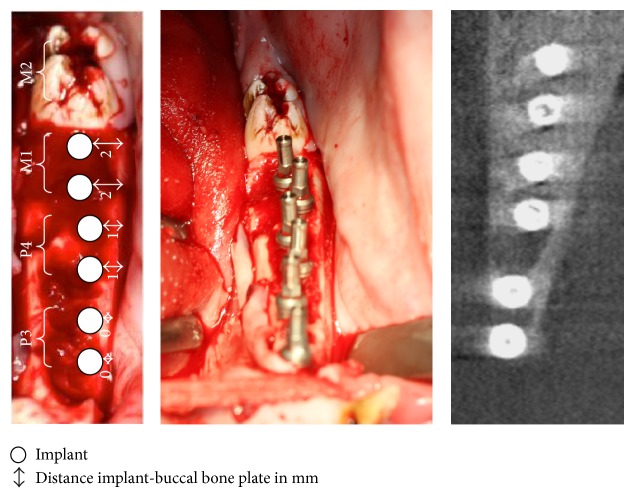
Clinical assessment and CBCT after surgery.

Thereafter a bilateral extraction of the first mandibular molar (M1) and two mandibular premolars (P3 and P4) was conducted, whereby the extraction was carried out atraumatically by piezosurgery (Piezosurgery 3, mectron, Cologne, Germany) with an osteotomy insert, a periotom, extraction levers, and pliers. Six implants were immediately placed in the extraction sockets on each side. The distance in between the implants was above 3 mm in order to prevent any influences on osseointegration. After extraction, the integrity of the buccal plate was checked with a periodontal probe. An implant bed for implants with regular platform (11.5 mm length, 4.1 mm diameter, Semados S-Line, BEGO, Bremen, Germany) was prepared according to manufactures guidelines and 6 implants on each side of the jaw were inserted. Implants were covered by a mucosal flap (see Figures 1 and 2).

To check the optimal clinical healing process, a follow-up examination in general anesthesia was conducted a week and a month after surgery. Wound dehiscences were recorded and if needed restitched after refreshing and cleaning of the edges with chlorhexidingluconate. Each implant loss was documented.

### 2.3. Animal Sacrifice, Biopsy Harvesting, and Processing

The peri-implant bone level was determined radiographically by CBCT images (SCS MedSeries® Verity H22e, SCS Software Computer Solutions GmbH; Aschaffenburg, Germany) with teeth in place, after extraction/before implantation and after implantation. After a 12-week healing period, all eight minipigs were sacrificed by intravenous applications of T61 (200 mg embutramide, 50 mg mebezonium, and 5 mg tetracaine per ml; 6 mg/50 kg) after deep sedation.

After euthanasia, the resection of the relevant sections of the jawbone were implemented with an oscillating bone saw. Standardized CBCT were made by the nonfixed tissue samples and morphometrically analyzed [24–26]. High Resolution CBCT (SCS MedSeries® Verity H22e, SCS Software Computer Solutions GmbH; Aschaffenburg, Germany) recordings were taken of all 8 minipigs. The SCS MedSeries Verity H22e was used due to specimens shape and size which lead to limitations in correct positioning within a stand-up device. It is usually intended to be used for X-ray computed tomography imaging of anatomies within upper and lower extremities. As the setting and specification of the X-ray tube and the flat panel detector match stand-up CBCT-scanners for imaging of the maxillofacial region, the results of measurements and image evaluation can be transferred. For the CBCT scans, the voxel size was set to 200 *μ*m in a standard scan protocol with a 193 × 242 mm field of view. Tube settings were 90 kV and 6 mA with 6 s exposure time. This setting was chosen in order to reflect clinical routine.

The specimens were separated from the surrounding tissue with a diamond band saw (EXAKT Apparatebau, Norderstedt, Germany) perpendicular to the buccal bone surface. Each tissue slice was documented by contact radiography (Faxitron, Tucson, USA). In order to produce the undecalcified histological specimen [27, 28], all samples were dehydrated using an ascending alcohol series (70%, 80%, 96%, and 100% ethanol), infiltrated with synthetic medium (Technovit 7200 VLC, Heraeus Kulzer, Wehrheim, Germany) and polymerized with blue light. After hardening, the specimens were processed with the cutting-grinding technique (Exakt, Norderstedt, Germany) to a ground section with a thickness of 30 *μ*m. No specimen was lost due to the procedure. After that, all specimens were stained with toluidine blue. Two independent observers (C. P. and J. M.) measured the thickness of the buccal bone perpendicular to the ninth implant thread and the distance from the implant shoulder to the ninth implant thread in the histologic ground section. Measurements were done with a microscope and defined magnification (50x) using an image analysis system (OsteoMeasure, OsteoMetrics, Decatur, USA) after calibration on test implants.

### 2.4. Radiologic Measurements in CBCT

**Figure 3:**
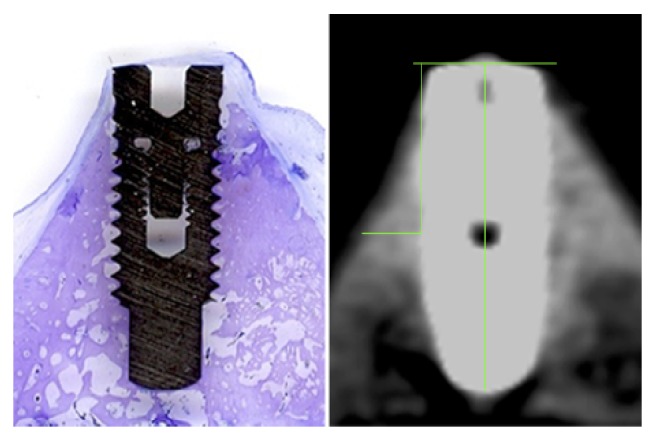
CBCT image with measurement lines and corresponding histologic slice specimen.

Altogether 60 implants could be evaluated in thin sections and CBCT images (for parameters, see Section 2.3). CBCT measurements were conducted in the software OsiriX MD (Version 3.0). Each implant was aligned in the same fashion and implants lengths were calibrated. The histologic measurements from implant shoulder to the ninth thread were transferred to the CBCT measurement software and the buccal bone thickness was measured individually by four different raters (J. S., oral surgeon; M. S., oral and maxillofacial surgeon; J. M., dentist; C. P., oral and maxillofacial surgeon; all are well trained reviewing CBCTs) (see Figure 3). The raters were blinded regarding the histologic measurement results.

### 2.5. Statistical Evaluation

Mean values and standard deviations were calculated for each parameter per animal. Random intercept models were used; variances and the Interclass Correlation Coefficient (ICC, Interrater Correlation) were calculated. The significance level was defined at *p* < 0,05. For assessing the agreement between CBCT and histology, we performed Bland-Altman plots. Mean differences ± 2 × standard deviation were computed [29]. All statistical analyses and plots were conducted with R Version 3.3.3 from the R-Project for statistical Computing (Vienna, Austria 2017) [30].

## 3. Results

Out of 96 inserted 36 implants had to be excluded from this study because histology and radiologic planes could not be aligned along the long axis of the implant due to the two using standardized protocols. Thus, CBCT images could be oriented in any direction in space, but not the histological sections accordingly. From 60 implants, bone thickness measurements in histology could be compared to bone thickness measurements in CBCT.

### 3.1. Mean Bone Thickness in Histology

**Figure 4:**
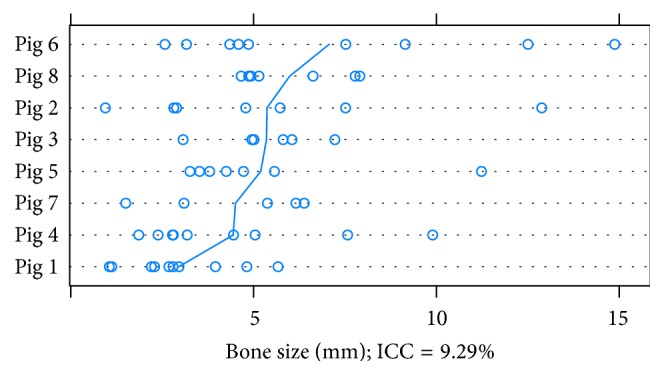
Dotplot for single values of bone size (mm) in descending order referring to mean value of the pig in histology. ICC: Intraclass Correlation Coefficient.

**Figure 5:**
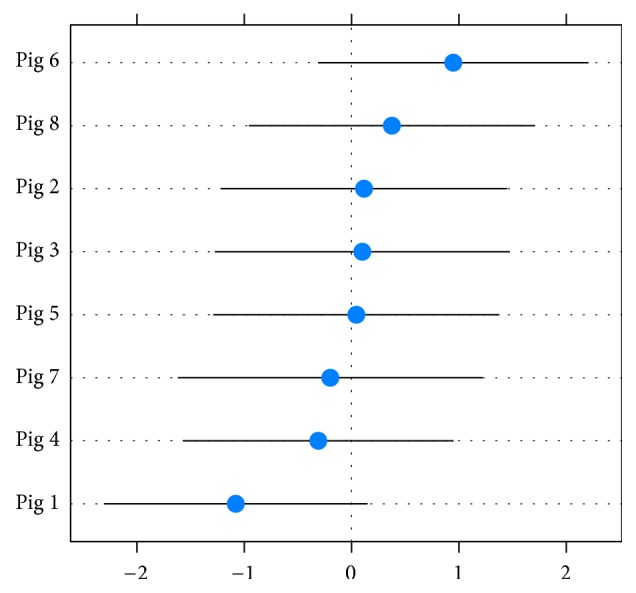
Distribution of random effects “pig.”

The mean bone thickness per implant measured in histology is pictured in Figure 4. The mean bone thickness was calculated in a random intercept model adjusted for correlated data due to the factor “pig.” The bone thickness was defined as fixed effect and the pig ID was the random effect. Controlled by the cluster “pig,” the mean of the bone thickness was 5.09 mm (CI 4.11–6.08 mm). The part of variance due to the pig was 9.29% (CI 0.00–25.14%) of total variance (Intraclass Correlation Coefficient (ICC)). The random effect was not significant (*p* value 0.02; see Figure 5).

### 3.2. Mean Bone Thickness in CBCT

**Figure 6:**
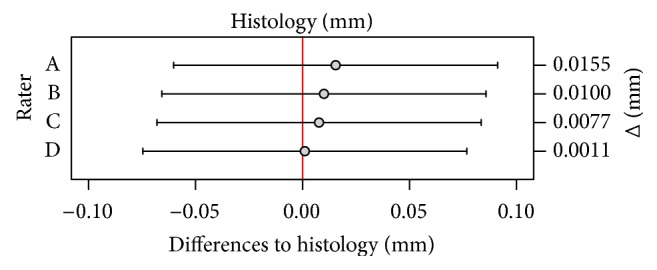
Means of bone thickness and confidence intervals measured by raters in CBCT compared to mean of bone thickness in histology.

Four random intercept models were calculated for the four raters with bone thickness in CBCT as fixed effect and pig ID as random effect. The mean bone thickness for each rater was as follows: Rater A: 5.10 mm (CI 4.12–6.10 mm); Rater B: 5.09 mm (CI 4.11–6.10 mm); Rater C: 5.09 mm (CI 4.09–6.12 mm); Rater D: 5.09 mm (CI 4.11–6.08 mm) (see Figure 6).

### 3.3. Differences of Bone Thickness in Histology and CBCT

**Figure 7:**
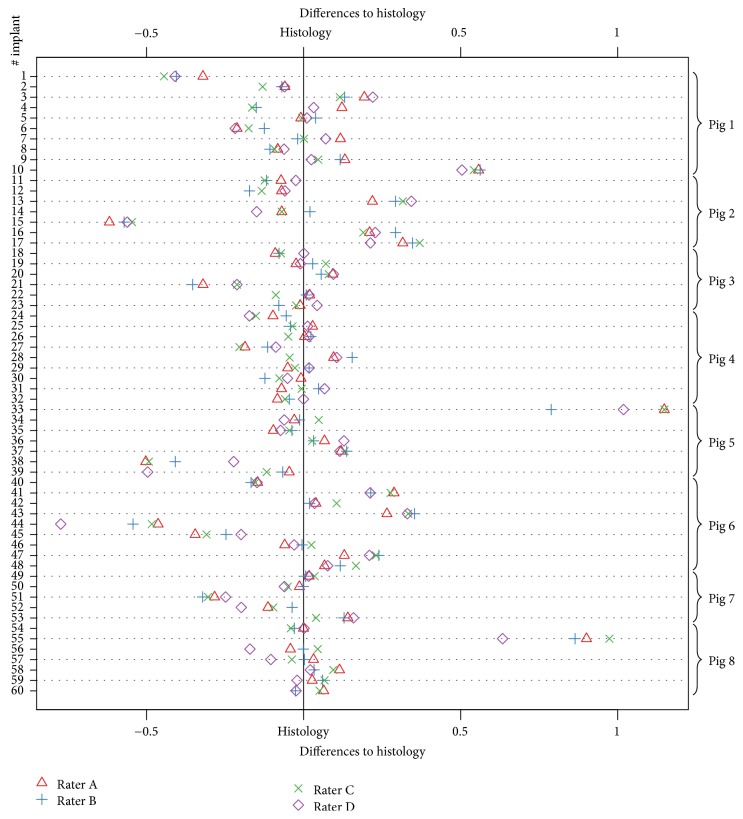
Differences of bone measurements in CBCT and histology (in mm).

**Figure 8:**
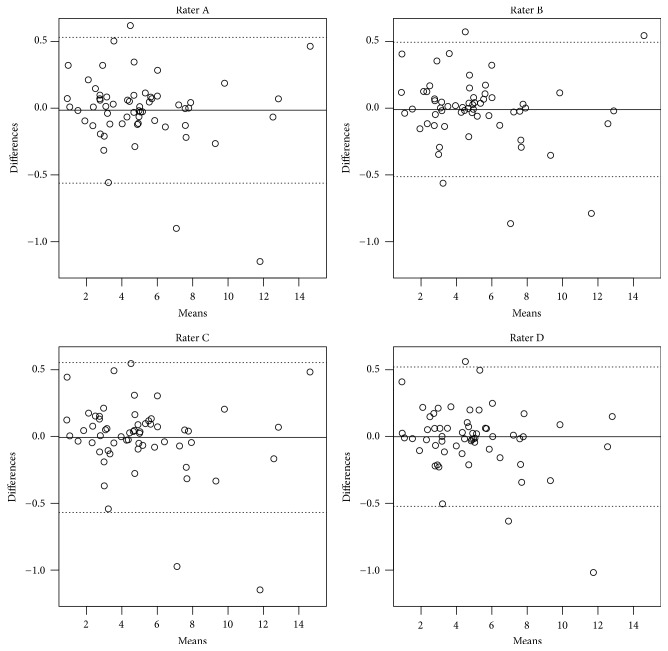
Bland-Altman plot: histology plotted against raters A–D.

The deviations of mean bone thickness measured by the raters in CBCT from the mean bone thickness in histology were marginal. All raters measured slightly more bone in CBCT than in histology (see Figures 6, 7, and 8). Averaged quarter millimeter more or less was measured compared to histology. The variance of pigs is compared to the variance of implants, not significant. The part of the variance due to rater was 1.35% of total variance (calculated without part of variance due to pig). The confidence interval (CI) was 0.00–3.01% of total variance. The random effect was not significant (*p* value 1.000). Therefore the Intraclass Correlation Coefficient (ICC, Interrater Correlation) was 98.65% (CI 100.00–96.99%). The Bland-Altman plot shows the range in bone thickness, plotted against the differences comparing histology and CBCT measurements for each rater (see Figure 8).

### 3.4. Correlation between Measurement Accuracy and Bone Thickness

To test the measurement accuracy in small bone sizes, the data set was grouped in “<3 mm” versus “≥3 mm” and “<2 mm” versus “≥2 mm.” A random intercept model was calculated while controlling for cluster effect “pig” and “rater.” The means of the groups ≥3 mm (0.033 mm) and ≥2 mm (0.019 mm) were slightly overestimated compared to histology, whereas the means of the groups <3 mm (−0.071 mm) and <2 mm (−0.099 mm) were slightly underestimated. The mean difference of each measurement compared to histology was in group <3 mm 0.152 mm, in group ≥3 mm 0.287 mm, in group <2 mm 0.170 mm, and in group ≥2 mm 0.271 mm. The ICC was in group <3 mm 99.32% (CI 100.00–91.42%), in group ≥3 mm 99.79% (CI 100–97.13%), in group <2 mm 97.47% (CI 100–83.45%), and in group ≥2 mm 100% (CI 100.00–97.35%).

**Figure 9:**
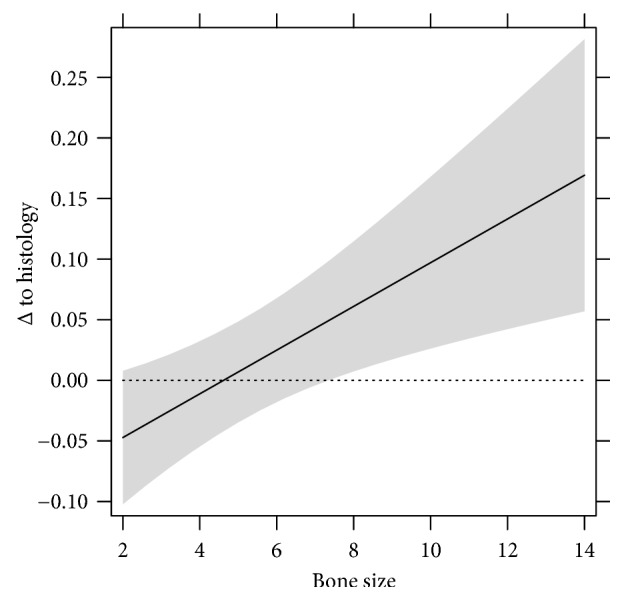
Difference of CBCT measurements to measurements in histology and influence of bone thickness (in mm) on average deviation.

An increase of one millimeter in bone thickness in CBCT leads to an additional average increase of measurement of 0.018 mm (CI 0.0006–0.030 mm). This effect is significant (*p* value 0.003) (see Figure 9).

## 4. Discussion

In this study, dental implants were inserted in pig jaws immediately after tooth extraction. Cone-beam computed tomographies were taken directly after scarification of the animals 6 months after surgery and the implants with surrounding bone tissue were processed histologically. The bone thickness directly around the implants was measured and compared correspondingly in histological sections and in CBCT datasets.

Bone thickness is crucial for a good initial implant stability and initial implant stability important for implant success [31]. Furthermore alveolar bone loss around implants over time is an important index for peri-implantitis and can lead to implant loss. For identification, the need of accurate measurement in CBCT datasets as a diagnostic tool is obvious. Most studies compared clinical assessment with radiologic measurement using different acquisition parameters and measurement tools [32–36]. CBCT was evaluated in staging periodontitis, determining alveolar bone defects and bone assessment around dental implants [32, 37–42]. Most of these studies stated, that CBCT images are adequate for the assessment of alveolar bone [21, 31, 35, 43, 44]. Nevertheless there are only a small number of studies comparing the CBCT measurements to histological measurements such as the present study. Ritter et al. placed 26 dental implants in dog jaws with chronic type vestibular defects and correlated CBCT measurements with histomorphometry of the vestibular bone level, oral bone thickness, and implant length. They found that 3D CBCT provides usable information about bone in all dimensions around implants with varying accuracy [43]. Wang et al. examined artificial defects in foxhounds after guided bone regeneration and implantation. They measured the bone thickness in a orobuccal dimension of 41 implants at different levels and thus evaluated the integration of different bone augmentation materials. Thereafter, peri-implant bone thickness could be measured at an accuracy of half a millimeter. The assessment of the existence and integration of bone augmentation material was partially possible [31]. In the present study, altogether 60 implants were evaluated by CBCT in correspondence to its histology. All raters measured slightly more bone in CBCT than in histology. By average, a quarter millimeter more or less was measured compared to histology. As voxel size was 0.2 mm in the present study, these findings are within expected technical limitations due to the given resolution and thus could be interpreted as technical or imaging artifacts.

Ritter et al. found an overestimation of about +0.3 mm (±0.04 mm) on average when comparing CBCT and histology. They stated that the direction of the implant artifacts is diagonal to the implant axis, which is why bone seems wider [43] as in reality. By forming groups (either ≥3 mm/<3 mm or ≥2 mm/<2 mm) in this study, smaller bone sizes are tending to be underestimated compared to wider bone sizes and higher bone sizes which were overestimated. An increase of one millimeter in bone thickness in CBCT increases the average measurement to additional 0.02 mm (CI 0.01–0.03 mm). This effect is significant (*p* value 0.003). González-Martín et al. presented a similar finding. For each millimeter of increase of bone thickness, the odds of radiographic identification increased by 30.6 (*p* < 0.001) [34]. Razavi et al. stated that above 1 mm of bone thickness, the accuracy of CBCT images increased substantially [45]. Dos Santos Corpas et al. found that the used CBCT deviates 1.20 mm from the histology when examining bone defects [38]. Fienitz et al. showed that CBCT is not accurate in sites of bone width lower than 0.5 mm [39]. The Interrater Correlation in the present and other studies was very high (98.65% (CI 100.00–96.99%)), indicating that CBCT is a reliable method for measuring bone structures [31, 35].

But, limitations in the present study are the use of CBCT scans obtained from isolated blocks of dead tissues and not from alive minipigs. Thus, CBCT datasets are representative of a best case scenario as CBCT has been shown at least for thin bone areas to be less accurate in living patients and different compared to what has been found in cadaver studies.

## 5. Conclusion

In this study there was no significant difference between CBCT measurement and corresponding histologic sections, indicating that CBCT is a valuable diagnostic tool for determining bone thickness in direct surrounding of dental implants without relevant artifacts. Thus, CBCT imaging is a reliable diagnostic tool to determine peri-implant bone loss and therefore estimate the risk of implant loss and the prognosis of implant survival. Another important clinical field could be the diagnosis of bone invasion from, for example, oral squamous cell carcinoma during tumor follow-up and staging without interference of CT specific metal artifacts.
